# Prophylactic Effects of Purple Shoot Green Tea on Cytokine Immunomodulation through Scavenging Free Radicals and NO in LPS-Stimulated Macrophages

**DOI:** 10.3390/cimb44090273

**Published:** 2022-09-02

**Authors:** Chih-Cheng Lin, Hsiu-Hua Lin, Hsiang Chang, Lu-Te Chuang, Chih-Yu Hsieh, Shing-Hwa Lu, Chi-Feng Hung, Jia-Feng Chang

**Affiliations:** 1 Department of Biotechnology and Pharmaceutical Technology, Yuanpei University of Medical Technology, Hsinchu 300, Taiwan; 2 Renal Care Joint Foundation, New Taipei City 220, Taiwan; 3 Department of Pet Healthcare, Yuanpei University of Medical Technology, Hsinchu 300, Taiwan; 4 Division of Urology, Department of Surgery, Taoyuan Branch of Taipei Veterans General Hospital, Taoyuan 330, Taiwan; 5 School of Medicine, Fu Jen Catholic University, New Taipei City 24205, Taiwan; 6 Department of Nursing, Yuanpei University of Medical Technology, Hsinchu 300, Taiwan; 7 Division of Nephrology, Department of Internal Medicine, Taoyuan Branch of Taipei Veterans General Hospital, Taoyuan 330, Taiwan; 8 School of Medicine, National Yang-Ming University, Taipei 120, Taiwan

**Keywords:** purple shoot green tea, antioxidant, free radicals, cytokine, prophylactic immunomodulation, polyphenol, flavonoid, tannin, proanthocyanidin, catechin, fermentation process

## Abstract

Polyphenols and flavonoids from non-fermented green tea and fully-fermented black tea exhibit antioxidant abilities that function as natural health foods for daily consumption. Nonetheless, evidence regarding prophylactic effects of purple shoot tea on immunomodulation remains scarce. We compared the immunomodulatory effects of different tea processes on oxidative stress and cytokine expressions in lipopolysaccharide (LPS)-stimulated macrophages. Major constituents of four tea products, Taiwan Tea Experiment Station No.12 (TTES No. 12) black and green tea and purple shoot black and purple shoot green tea (TB, TG, PB and PG, respectively), were analyzed to explore the prophylactic effects on expressions of free radicals, nitric oxide (NO), monocyte chemoattractant protein-1 (MCP-1), interleukin 6 (IL-6) and tumor necrosis factor α (TNF-α) in LPS-activated RAW264.7 cell models. PG contained abundant levels of total polyphenols, flavonoids, condensed tannins and proanthocyanidins (371.28 ± 3.83; 86.37 ± 1.46; 234.67 ± 10.1; and 24.81 ± 0.75 mg/g, respectively) contributing to excellent free radical scavenging potency. In both the LPS-activated inflammation model and the prophylactic model, all tea extracts suppressed NO secretion in a dose-dependent manner, especially for PG. Intriguingly, most tea extracts enhanced expressions of IL-6 in LPS-stimulated macrophages, except PG. However, all teas disrupted downstream transduction of chemoattractant MCP-1 for immune cell trafficking. In the prophylactic model, all teas inhibited inflammatory responses by attenuating expressions of IL-6 and TNF-α in a dose-dependent manner, especially for TG and PG. Our prophylactic model demonstrated PG exerts robust effects on modulating LPS-induced cytokine expressions of MCP-1, IL-6 and TNF-α through scavenging free radicals and NO. In light of the prophylactic effects on LPS-related inflammation, PG effectively scavenges free radicals to modulate cytokine cascades that could serve as a functional beverage for immunomodulation.

## 1. Introduction

Inflammation is a protective response to microbial invasion, various toxins and oxidative injury as the defense system of hosts against pathogens, whilst persistent inflammation caused by an uncontrolled infection can produce lethal levels of pro-inflammatory mediators that contribute to tissue destruction and multi-organ failure [1]. Such dysregulated inflammatory misfiring results in excessive chemoattractants and subsequent reactive oxygen species (ROS) that play major roles in the pathogenesis of severe acute respiratory syndrome, e.g., uremic lung injury and pneumonia [2,3,4]. Increased inflammatory cytokines, such as interleukin-6 (IL-6) and tumor necrosis factor alpha (TNF-α), are indeed observed in patients with severe pneumonia [5]. Research indicates that the cytokine storms in severe pneumonia may be involved in high levels of interleukin (IL)-6 and monocyte chemoattractant protein-1/C-C motif chemokine ligand 2 (MCP-1/CCL2) [6,7]. In view of the evidence above, foods with immunomodulatory functions targeting ROS, TNF-α, IL-6 and MCP-1 could serve as adjunctive therapy to reduce the occurrence of cytokine storms while facing bacterial infection [6,8,9].

The anti-inflammatory and antioxidant bioactivities in functional foods were discovered to be closely correlated with major constituents and phytochemical composition [10,11,12,13,14,15]. Prompt inhibition of nitric oxide (NO) production in lipopolysaccharide (LPS)-stimulated macrophages is an important strategy for the treatment of systemic bacterial infection [11]. Natural plant extracts containing polyphenols, flavonoids, tannins and proanthocyanidins could be considered as beneficial agents for improving the disease progression, e.g., anti-degenerative, anti-rancidity, anti-cancer, anti-infectious, anti-allergic, anti-viral, anti-mutagenic, wounding healing, cardio-protective and anti-diabetic effects [16,17,18]. Tea, a popular functional beverage, contains high levels of the abovementioned bioactive compounds that exhibit prominent immunomodulatory [19,20] and antioxidant effects on preventing infectious diseases [21]. Myriads of studies have shown that tea polyphenols may contribute to reducing inflammation through scavenging free radicals and modulating cytokine cascades [22]. Catechins have been proved to exert their anti-fibrotic effect and downregulate signaling pathways of proinflammatory mediators that functions as supplements to counteract overwhelmed inflammatory burden in COVID-19 patients [23,24]. In addition to the catechins, emerging evidence points out that the contents of anthocyanidins and anthocyanins possess not only antioxidant activities but also anti-microbial effects. A novel bred cultivar of tea clones with purple-colored buds and leaves in Taiwan, purple-shoot tea, has been found which contains extremely high levels of total polyphenols, flavonoids, anthocyanidins and anthocyanins, contributing to prominent antioxidant and anti-inflammatory properties [25]. Thus, we developed a prophylactic model to investigate the antioxidant effects of different fermentation processes on cytokine-related inflammatory signaling pathways in LPS-stimulated macrophages by analyzing major constituents of various teas, ROS/NO scavenging abilities and cytokine modulation (IL-6/MCP-1/TNF-α) in the present study.

## 2. Materials and Methods

### 2.1. Tea Preparation and Fermentation

Two varieties of cultivated tea clones, Purple-Shoot Tea 113 (registration in progress) and Taiwan Tea Experiment Station (TTES) No. 12, were selected for different fermentation processes: non-fermented green tea and fully-fermented black tea. Young tender shoots comprising of two unfolded younger leaves with a bud were hand harvested. These two kinds of tea varieties, processed to both green tea and black tea, were kindly provided by the Tea Research and Extension Station (Taoyuan, Taiwan). All of the tea was milled into powder using a grinder. The powder samples were extracted twice with a 20-fold volume of water for 60 min while vigorously shaking. After the samples were filtered using Whatman No.1 filter papers, the filtrates were dehydrated using a freeze-dryer. The extracts of TTES No. 12 black/green tea and purple shoot black/green tea were defined as TB, TG, PB and PG, respectively.

RAW 264.7 macrophage cell line was obtained from the Bioresource Collection and Research Center (Hsinchu, Taiwan). The cells were cultured in DMEM medium supplemented with 10% heat-inactivated fetal bovine serum and maintained at 37 ℃ in a humidified incubator containing 5% CO_2_. LPS of bacterial endotoxins, 3-(4,5-dimethylthiazol-2-yl)-2,5-diphenyl-tetrazolium bromide (MTT), Folin-Ciocalteu reagent and BCIP/NBT liquid substrate system were purchased from Sigma–Aldrich Corp. (St. Louis, MO, USA). Commercial quantitative enzyme-linked immunosorbent (ELISA) assays for IL-6 (Catalog Number: 88-7364) and TNF-α (Catalog Number: 88-7324) were purchased from ThermoFisher Scientific Inc. (Waltham, MA, USA). A MCP-1/CCL2 ELISA kit (Catalog Number: RAB0056) were purchased from Sigma–Aldrich Corp. (St. Louis, MO, USA). All other chemicals were of analytical purity grade.

### 2.2. Characterization of Phytochemicals

**Figure 1:**
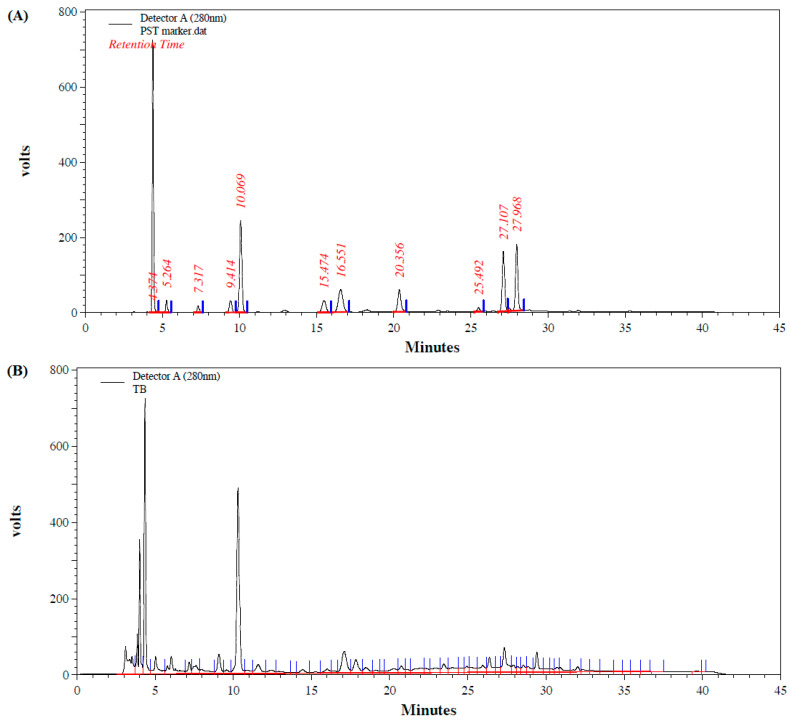

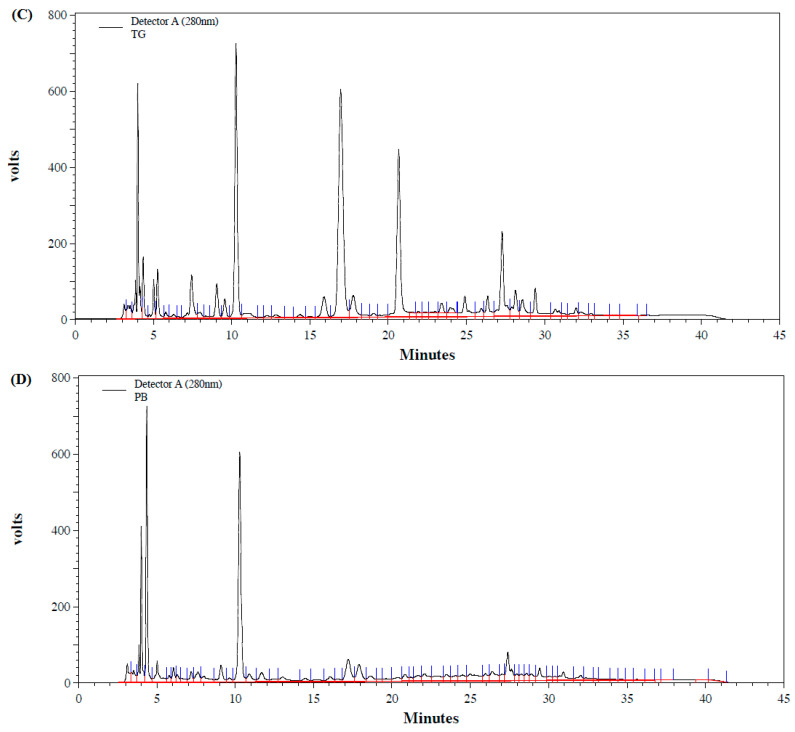

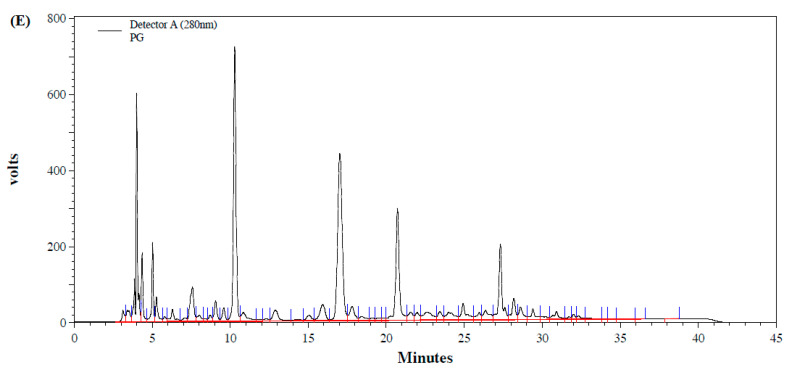
The high-performance liquid chromatography system to analyze the major constituents in various tea extracts. The major constituents in various tea extracts were determined by comparing their retention times to authentic standards in the high-performance liquid chromatography analysis. (**A**) standards; (**B**) TB; (**C**) TG, (**D**) PB and (**E**) PG. Abbreviations: T: Taiwan Tea Experiment Station No. 12; P: purple shoot tea; G: green tea; B: black tea.

The total polyphenols (mg gallic acid eq./g), total flavonoids (mg catechin eq./g), condensed tannin (mg catechin eq./g) and anthocyanidin (mg cyanidin eq./g) presenting in the tea extracts were determined using high-performance liquid chromatography (HPLC) (Shimadzu, Tokyo, Japan) [26]. The operating parameters were as follows: column: C18; mobile phase: solvent A—water-acetic acid (25:1), solvent B—methanol; pumps (Binary Gradient); T.Flow: 1.000 mL/min; P.Max: 400.0 kgf/cm^2^; P.Min: 0.0 kgf/cm^2^; CTO-10 ASvp, temperature: 40 °C; SPD-10 Avp (Det.A): lamp: D2 and polarity: +Ve. The data were analyzed using Shimadzu Class-VP software (Version 6.14 SP2). Figure 1 showed the identification by comparing their retention times to authentic standards of the chromatographic analysis (A): standards; (B): TB; (C): TG, (D): PB and (E): PG.

### 2.3. Scavenging Ability on DPPH Scavenging Ability

The 2,2-diphenyl-1-picryhydrazyl (DPPH) radical-scavenging capacity of the tea extracts was determined for antioxidant activity. Serially diluted samples of the tea extracts were pipetted into a 96-well plate. Next, DPPH methanolic solution was added into each well, and the plate was shaken for 5 min. The change in absorption at 540 nm after the addition of DPPH was measured using an enzyme-linked immunosorbent assay reader (EL800, Bio-Tek Instruments Inc., Winooski, VT, USA).

### 2.4. Scavenging Ability on Nitrite Oxide

The scavenging abilities of tea extracts on NO were measured according to the method previously described in our research [20]. A total of 4 mL of sample solution was added to 1 mL of 25 mM sodium nitroprusside solution and incubated at 37 °C for 150 min. An aliquot of the incubation solution was removed and diluted with 0.3 mL of Griess regent (1% sulfanilamide in 5% H_3_PO_4_ and 0.1% naphthylethylenediamine dihydrochloride). The absorbance of the chromophore was read at 575 nm and referred to the absorbance of standard solutions of sodium nitrite salt treated in the same way with Griess reagent.

### 2.5. Determination of NO Production and Cytokine Expressions

To determine the effect of tea extracts on the production of NO, the RAW 264.7 cells were cultured at a density of 1 × 10^5^ cells per well in a 96-well culture plate. Following a 4-h incubation, the adherent cells were washed 3 times with PBS. In the inflammatory model, the cells were incubated in medium with different doses of tea samples (0.2, 0.3, 0.4 and 0.5 mg/mL) and 0.5 μg/mL LPS for 24 h until assay. In our prophylactic model, the cells were pretreated with different doses of tea samples (0.2, 0.3, 0.4 and 0.5 mg/mL) for 8 h and LPS was co-incubated for the remaining 16 h to explore the prophylaxis of the teas. Moreover, cell viability was evaluated using the MTT method. Finally, nitrite concentration in the medium was measured as an indicator of NO production using the Griess reaction [20]. The levels of cytokines (IL-6, TNF-α) and chemokines (MCP-1/CCL2) in the culture medium were measured using an enzyme-linked immunosorbent assay (ELISA). IL-6 (catalog number: 88-7364) and TNF-α (catalog number: 88-7324) were purchased from ThermoFisher Scientific Inc. (Waltham, MA, USA). MCP-1/CCL2 ELISA kits (catalog number: RAB0056) were purchased from Sigma–Aldrich Corp. (St. Louis, MO, USA). Above measurements were in accordance with the manufacturer’s instructions.

### 2.6. Statistical Analysis

All data are presented as mean ± SE using GraphPad Prism 8 (GraphPad Software, Inc., San Diego, CA, USA) or SPSS version 22.0 (IBM, Armonk, NY, USA). Quantitative data from three independent experiments were analyzed using ANOVA and Tukey—Kramer’s tests. A *p*-value < 0.05 was considered statistically significant for each of the experiments.

## 3. Results

### 3.1. Phytochemicals Content Analyses

To study the relationship between the major constituents for and antioxidant/anti-inflammatory activity in different tea extracts, the proximate analysis of various phytochemical compounds were identified. Total polyphenols, flavonoids, condensed tannins and proanthocyanidins from four tea extracts are shown in Table 1. Overall, PG contained the highest concentrations of polyphenols, flavonoids, condensed tannins, and proanthocyanidins than other tea extracts (TG, TB and PB). However, the purple shoot teas (TG and TB) appeared to contain significantly more total flavonoids and proanthocyanidins than TTES No. 12 teas (TG and TB). As expected, results were similar to those in our previous study in 2012 [25]. It is interesting that the concentration of proanthocyanidins in purple shoot tea (PG and PB) was twice than TTES No. 12 teas (TG and TB). The results of chemical composition analysis showed that the levels of condensed tannins in PB and TB were not statistically different, but the green teas (PG and TG) were significantly higher than those of the black teas (PB and TB).

The identifications of diverse types of catechins were determined using HPLC according to the retention times obtained from authentic standards run under identical conditions. The major constituents of catechins in both tea leaves include: (-)-epigallocatechin gallate (EGCG), (-)-epigallocatechin (EGC), (-)-gallocatechin-3-*gallate* (GCG) and gallocatechin (GC), with EGCG being the most abundant catechins in the tea (Table 2). All of the contents, the various types of catechins in the green teas were higher than in the black teas, except for gallic acid. Overall, purple shoot teas contained more abundant catechins than TTES No. 12 teas, which is similar to the results of polyphenols in Table 1.

### 3.2. Antioxidant Activity via DPPH and NO Radical Scavenging Assays

**Figure 2:**
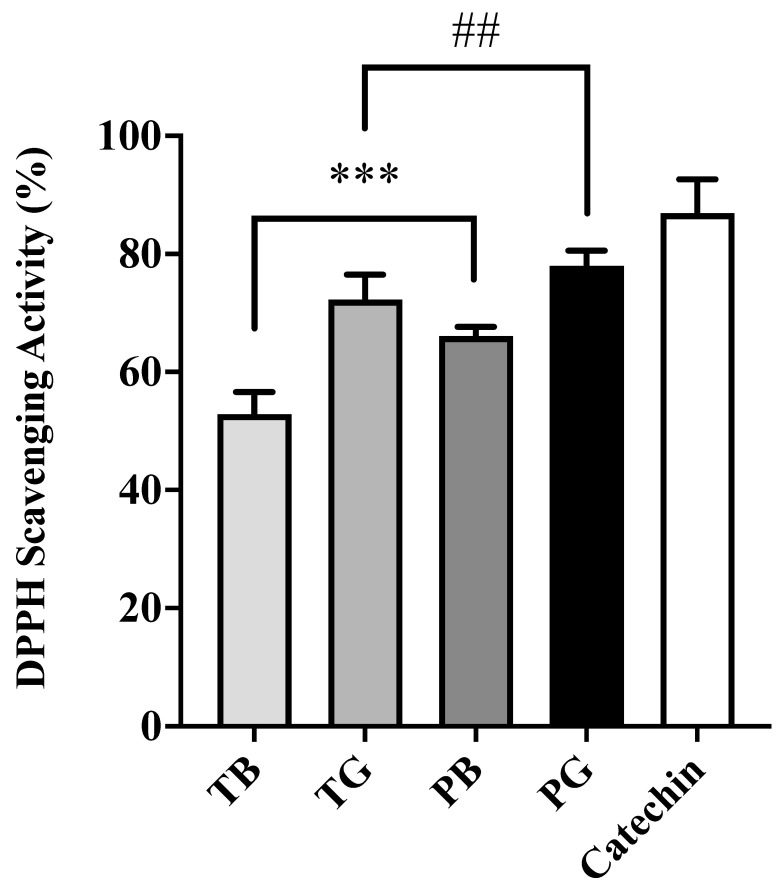
The scavenging ability of DPPH radicals of four tea extracts compared with the standard catechin as reference. The data were presented as the mean ± S.E. of three independent experiments. ## *p* < 0.01 and *** *p* < 0.001. Note that PG exerted the highest DPPH scavenging ability that was superior to catechin. T: Taiwan Tea Experiment Station No. 12; P: purple shoot tea; G: green tea; B: black tea and DPPH: 1,1-diphenyl-2-picrylhydrazyl.

**Figure 3:**
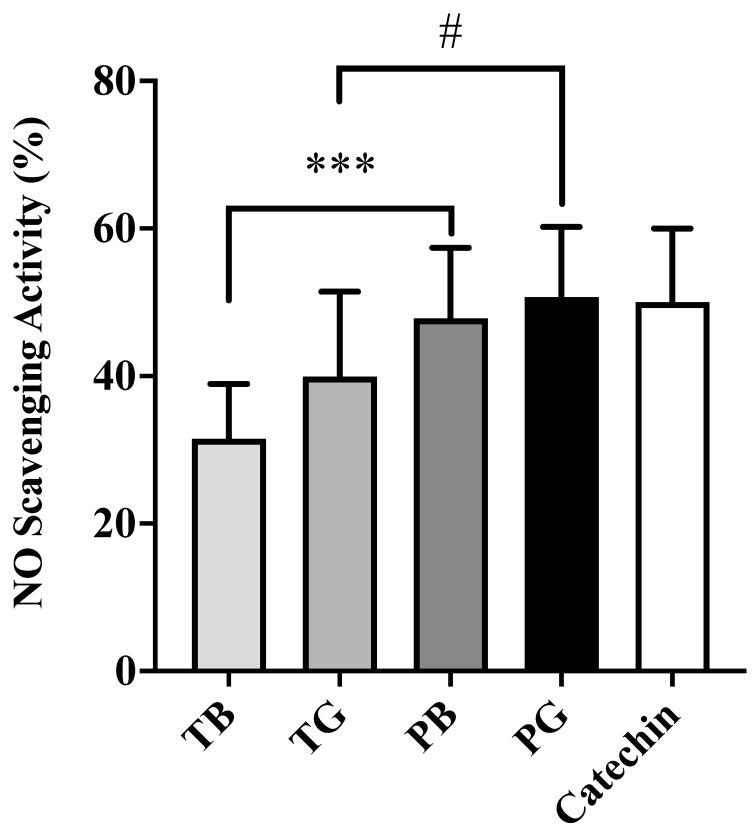
The scavenging ability of NO radicals of four tea extracts compared with the standard catechin as reference. The data were presented as mean ± S.E. of three independent experiments. # *p* < 0.05 and *** *p* < 0.001. Note that PG exerts the highest NO scavenging ability that was superior to catechin. NO: Nitric oxide; T: Taiwan Tea Experiment Station No. 12; P: purple shoot tea; G: green tea and B: black tea.

To investigate the relationship between chemical constituents and antioxidant activity, purple shoot tea extracts rich in flavonoids and proanthocyanidins were compared with TTES No. 12 tea extracts and the standard catechin as the reference. The DPPH and NO radical scavenging abilities were used in this study to evaluate antioxidant abilities. As shown in Figure 2, the scavenging potencies of green tea (TG and PG) exhibited more powerful DPPH scavenging activity than black tea (TB and PB). In general, purple shoot teas have a higher antioxidant capacity than TTES No. 12 teas. The DPPH scavenging effects of four tea extracts are concentration-dependent, with total polyphenols and catechins similar to our prior research results [26]. As shown in Figure 3, four tea extracts were also evaluated for their scavenging effects on NO radicals along with the standard catechin as the reference. The scavenging effects of four tea extracts are in a dose-dependent manner. Likewise, purple shoot teas exert a strong antioxidant effect for which the order of potency for NO eradication reveals PG > PB > TG > TB. Intriguingly, the NO scavenging ability of PG (52.8%) was equivalent to pure catechin compound (50.9%). Proanthocyanidins are comprehensively defined as oligomers flavonoids built up from polyhydroxy flavan-3-ol units. Total flavonoids and oligomeric proanthocyanidins might be related to the NO scavenging ability in the aqueous-phase analytical system [20]. The NO scavenging result is slightly different to DPPH scavenging ability. That is, flavonoid- and proanthocyanidin-rich purple shoot tea (PG and PB) possesses higher NO radical scavenging ability than TTES No. 12 (TG and TB), even PB is higher than TG.

### 3.3. Dose Effects of Various Tea Extracts on Inhibiting NO Production in LPS-Stimulated Macrophages

**Figure 4:**
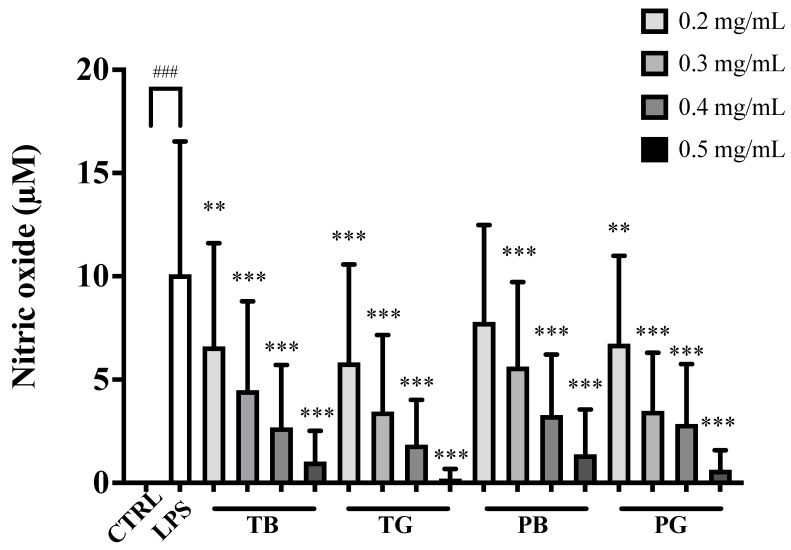
Effects of various concentrations of tea treatments on nitrite inhibition rates in LPS-stimulated macrophages as the inflammatory model. LPS (0.5 μg/mL) was added to all groups at time 0 h for 24 h until assay, except the control group. In the LPS-stimulated macrophages, the cells were incubated in the medium without/with various concentrations of tea samples. The data were presented as mean ± SE of three experiments. ** *p* < 0.01, *** *p* < 0.001 and ^###^ *p* < 0.001. LPS: Lipopolysaccharide. T: Taiwan Tea Experiment Station No. 12; P: purple shoot tea; G: green tea and B: black tea.

**Figure 5:**
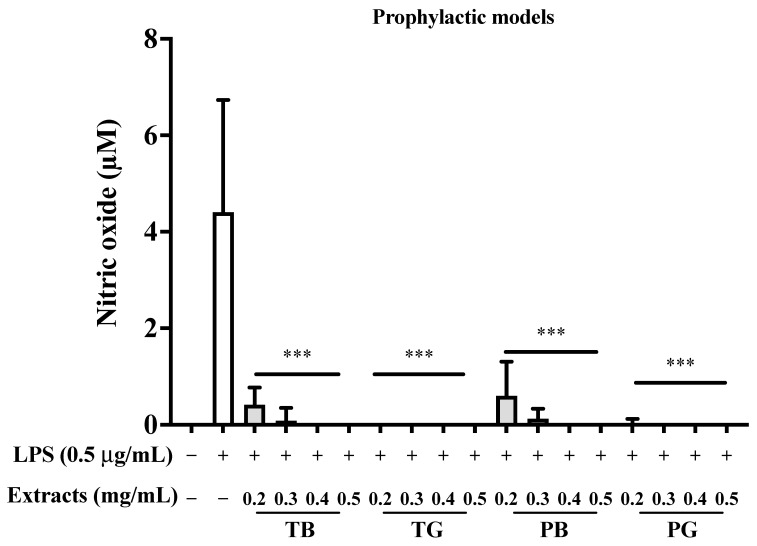
Effects of various concentrations of tea pretreatments on nitric oxide inhibition rates in LPS-stimulated macrophages as the prophylactic model. In our prophylactic model, the pretreatment of four tea extracts was performed for 8 h. Then LPS (0.5 μg/mL) was added and co-incubated for the remaining 16 h to explore the prophylaxis of the four teas. The data were presented as mean ± SE of three experiments. *** *p* < 0.001. LPS: Lipopolysaccharide. T: Taiwan Tea Experiment Station No. 12; P: purple shoot tea; G: green tea and B: black tea.

In view of the above concentration-dependent ROS scavenging effects, we aim to explore the optimal therapeutic dose of four tea extracts. Analysis of NO production by measuring the nitrite with the Griess reaction revealed that placing unstimulated RAW 264.7 cells in culture medium for 24 h produced a basal amount of nitrite. When the cells were incubated with tea extracts in the absence of LPS, the nitrite concentrations in the medium were maintained at a background level similar to that in the unstimulated control (data not shown). RAW 264.7 cells stimulated with LPS for 24 h were defined as our inflammatory model. After treatment with LPS for 24 h, the nitrite concentrations in the medium were markedly increased compared with the control group (*p* < 0.001). In the inflammatory model, significant inhibition of NO production was detected when cells were cotreated with LPS and various concentrations of tea extracts in a dose-dependent manner (Figure 4). In contrast, macrophages pretreated with tea samples for 16 h prior to LPS stimulation serve as our prophylactic model (Figure 5). All tea extracts exhibit higher inhibition rates in the prophylactic model than in the inflammatory model, as shown in Figure 5. In addition, the inhibition of four tea extracts on NO in LPS-stimulated RAW264.7 cells showed that green tea was better than black tea, particularly in the prophylactic model. Current data were in accordance with the above results of antioxidant activity. These findings suggested that purple shoot teas could act as prophylactics by increasing ROS elimination in the LPS-stimulated RAW 264.7 cells, especially for PG.

### 3.4. Expressions of Cytokines, TNF-α, IL-6 and MCP-1/CCL2 in the Inflammatory Model

**Figure 6:**
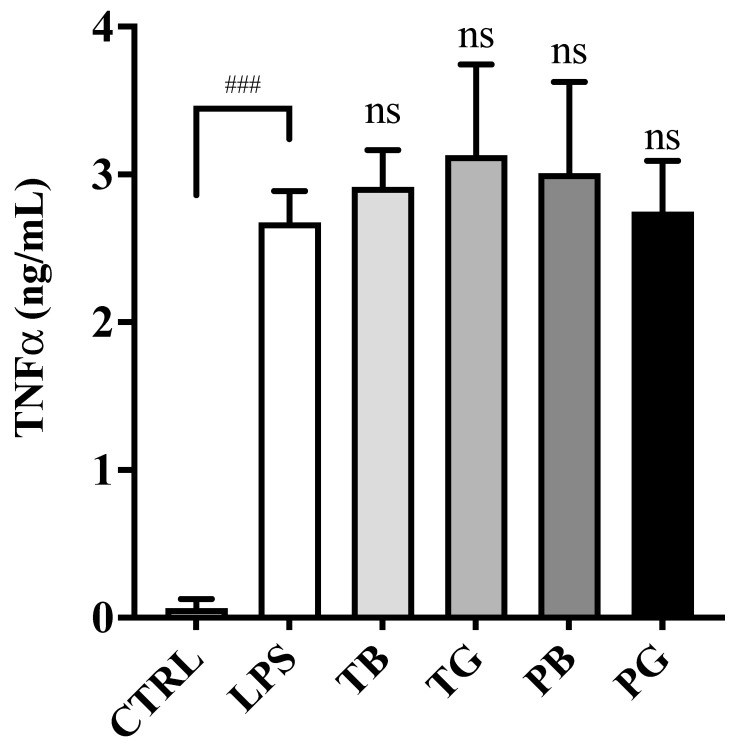
Effects of various tea treatments on TNF-α secretion in LPS-stimulated macrophages as the inflammatory model. LPS (0.5 μg/mL) was added to all groups at time 0 h for 24 h until assay, except the control group. In the LPS-stimulated macrophages, the cells were incubated in the medium without/with tea samples (0.5 mg/mL). The data were presented as mean ± SE of three experiments. ### *p* < 0.001 vs. control; ns *p* > 0.05 vs. LPS. LPS: Lipopolysaccharide; T: Taiwan Tea Experiment Station No. 12; P: purple shoot tea; G: green tea and B: black tea. TNF-α, tumor necrosis factor-α.

**Figure 7:**
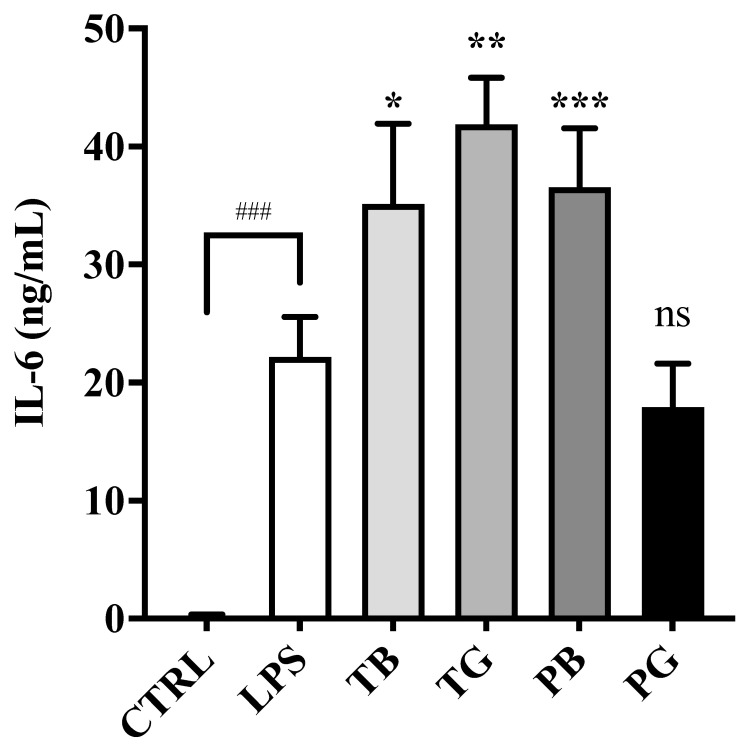
Effects of various tea pretreatment on IL-6 secretion in LPS-stimulated macrophages as the inflammatory model. LPS (0.5 μg/mL) was added to all groups at time 0 h for 24 h until assay, except the control group. In the LPS-stimulated macrophages, the cells were incubated in the medium without/with tea samples (0.5 mg/mL). The data were presented as mean ± SE of three experiments. * *p* < 0.05; ** *p* < 0.01; *** *p* < 0.001, and ns *p* > 0.05 vs. LPS group and ### *p* < 0.001 vs. control. IL-6: Interleukin-6; LPS: lipopolysaccharide; T: Taiwan Tea Experiment Station No. 12; P: purple shoot tea; G: green tea and B: black tea.

**Figure 8:**
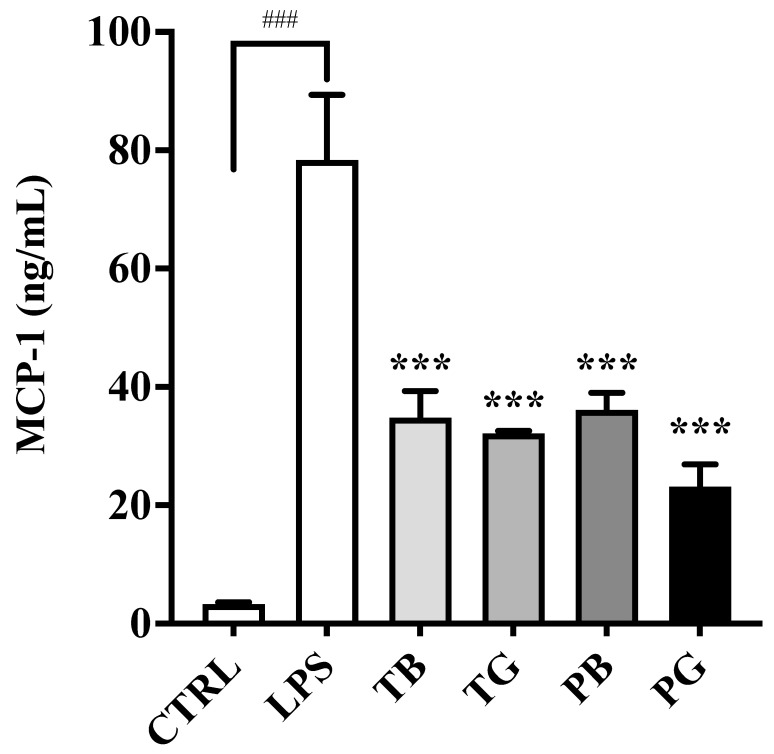
Effects of various tea treatments on MCP-1 secretion in the LPS-stimulated macrophages as the inflammatory model. LPS (0.5 μg/mL) was added to all groups at time 0 h for 24 h until assay, except the control group. In the LPS-stimulated macrophages, the cells were incubated in the medium without/with tea samples (0.5 mg/mL). The data were presented as mean ± SE of three experiments. *** *p* < 0.001 vs. LPS group and ### *p* < 0.001 vs. control. MCP-1: Monocyte chemoattractant protein-1; LPS: lipopolysaccharide; T: Taiwan Tea Experiment Station No. 12; P: purple shoot tea; G: green tea and B: black tea.

Recent research suggested that the inflammation cascade in severe COVID-19 patients with respiratory failure are intricately associated with an increase in TNF-α [5], IL-6 and MCP-1 [6]. In the absence of tea extract pretreatments, the macrophages stimulated with LPS significantly increased the secretion of TNF-α (*p* < 0.001) (Figure 6), IL-6 (*p* < 0.001) (Figure 7) and MCP-1/CCL2 (*p* < 0.001) (Figure 8). While LPS-stimulated macrophages were treated with tea extracts at time 0 h, all tea extracts at the concentration of 0.5 mg/mL showed no inhibitory effects on expression of the TNF-α and IL-6 in the inflammatory model (Figure 6 and Figure 7). Intriguingly, most teas might slightly enhance expressions of IL-6 in the inflammatory model, except PG. Of great importance, TB, TG, PB and PG suppressed the secretion of MCP-1/CCL2 at a concentration of 0.5 mg/mL compared to the control groups in the inflammatory model (all *p* values < 0.001) (Figure 8). PG showed the highest inhibitory activity on MCP-1/CCL2. The expression of pro-inflammatory cytokines (MCP-1/CCL2) produced by LPS-stimulated macrophages was significantly and dose-dependently attenuated by all tea extracts, suggesting that ROS-induced cytokines and chemoattractants involved in the pathogenesis of inflammatory cascades were more complex than illustrated (Figure 6, Figure 7 and Figure 8).

### 3.5. Inhibition of TNF-α, IL-6 and MCP-1/CCL2 in the Prophylactic Model

**Figure 9:**
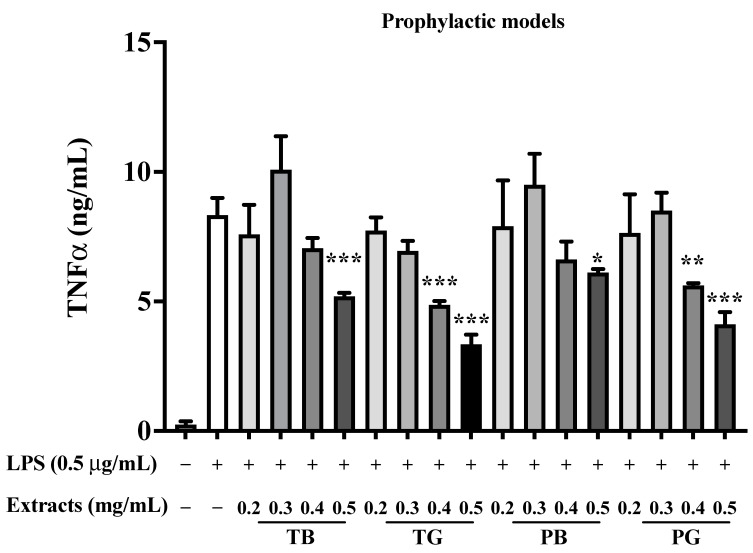
Effect of various teas on TNF-α secretion of LPS-stimulated macrophages in the prophylactic model. LPS (0.5 μg/mL) was added to all groups until assay, except the control group. In the LPS-stimulated macrophages, the cells were incubated in the medium without/with various concentrations of tea samples. In our prophylactic model, the pretreatment of four tea extracts was performed for 8 h. Then LPS (0.5 μg/mL) was added and co-incubated for the remaining 16 h to explore the prophylaxis of the four teas. The data were presented as mean ± SE of three experiments. * *p* < 0.05; ** *p* < 0.01; *** *p* < 0.001. TNF-α: tumor necrosis factor-α; LPS: lipopolysaccharide; T: Taiwan Tea Experiment Station No. 12; P: purple shoot tea; G: green tea and B: black tea.

**Figure 10:**
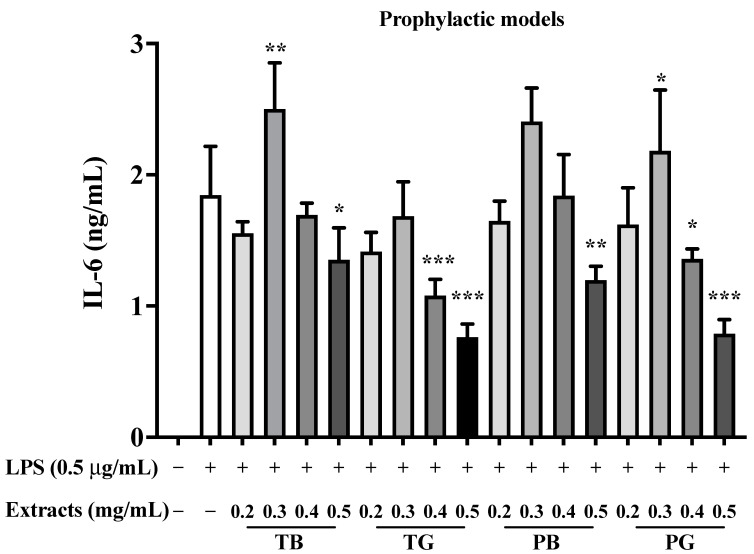
Effect of various extracts from tea on IL-6 secretion of LPS-stimulated macrophages in the prophylactic model. LPS (0.5 μg/mL) was added to all groups until assay, except the control group. In the LPS-stimulated macrophages, the cells were incubated in the medium without/with various concentrations of tea samples. In our prophylactic model, the pretreatment of four tea extracts was performed for 8 h. Then LPS (0.5 μg/mL) was added and co-incubated for the remaining 16 h to explore the prophylaxis of the four teas. The data were presented as mean ± SE of three experiments. * *p* < 0.05; ** *p* < 0.01; *** *p* < 0.001. IL-6: Interleukin-6; LPS: lipopolysaccharide; T: Taiwan Tea Experiment Station No. 12; P: purple shoot tea; G: green tea and B: black tea.

In the prophylactic model, the expression of TNF-α and IL-6 induced by LPS-stimulated macrophages was attenuated by the four tea extracts in a dose-dependent manner. More specifically, TB, TG, PB, and PG robustly reduced the secretion of TNF-αat a concentration of 0.5 mg/mL compared to the control groups (all *p* values < 0.001), respectively (Figure 9), respectively. Furthermore, the expressions of IL-6 secreted by LPS-stimulated macrophages were all significantly inhibited at a concentration of 0.5 mg/mL compared to the control groups in our prophylactic model (all *p* values < 0.001), respectively (Figure 10). The results revealed that green tea extracts (TG and PG) processed higher down-regulated effects on LPS-induced TNF-α and IL-6 expressions than black tea extracts (TB and PB) in accordance with our previous research [20].

## 4. Discussion

Both green tea or black tea extracted from Purple Shoot Tea (PG and PB) and TTES No.12 tea (TG and TB) exhibited various degrees of antioxidant and anti-inflammatory effects. Moreover, such antioxidant and anti-inflammatory activities were indeed robust in our prophylactic model. In a dose-dependent manner, the downregulation of effects on inflammatory signaling pathways of tea extracts were found to be tightly correlated with antioxidant activity and polyphenol concentrations in the prophylactic model. Nonetheless, the therapeutic effects of administration of tea extracts after LPS-activated macrophage inflammation may be insignificant, suggesting that tea extracts could not act as therapeutic agents following bacterial infection. In accordance with a myriad of prior research, we proved that natural tea products could act as preventive agents against many LPS inflammation-related diseases [19,20,21,22,24]. Our four tea extracts could eradicate free radicals to suppress pro-inflammation mediators through the modulation of MCP-1, TNF-αand IL-6 in the prophylactic model. According to a recent large-sample clinical study with 100,902 participants published in the European Journal of Preventive Cardiology, drinking tea at least three times a week is associated with, not only a longer life expectancy, but also, lower risks of cardiovascular disease and all-cause death [27]. Concerning the extremely high prevalence of sudden death and silent hypoxia during the COVID-19 era, habitual tea consumption may exert multifaceted health benefits against premature death.

**Figure 11:**
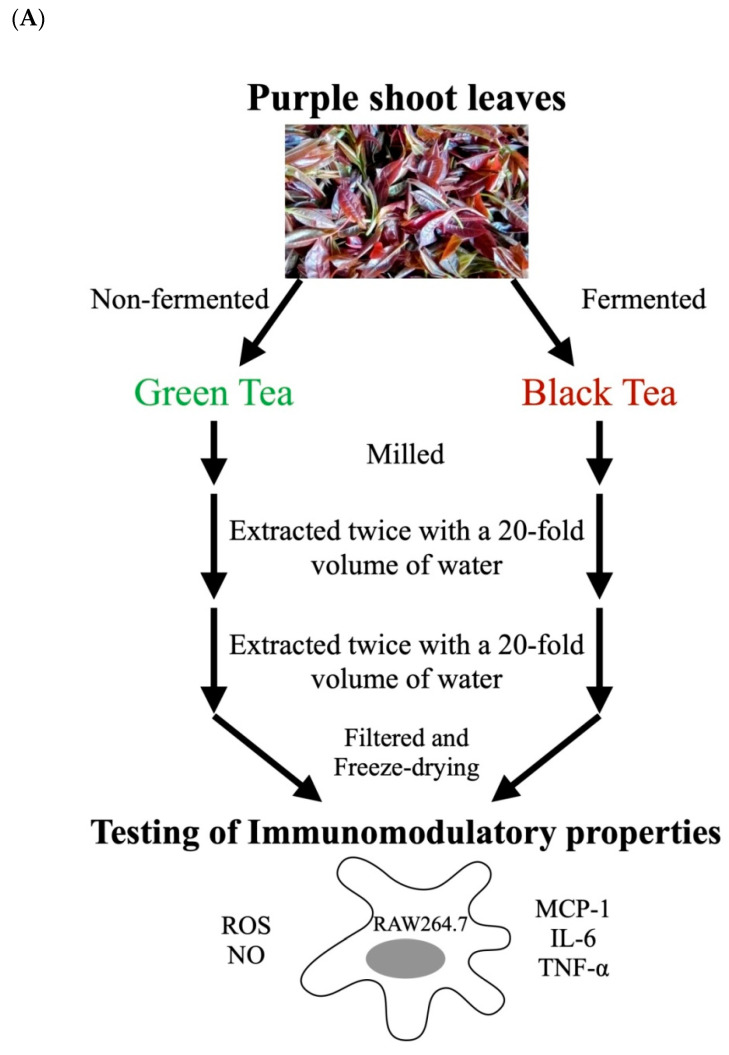

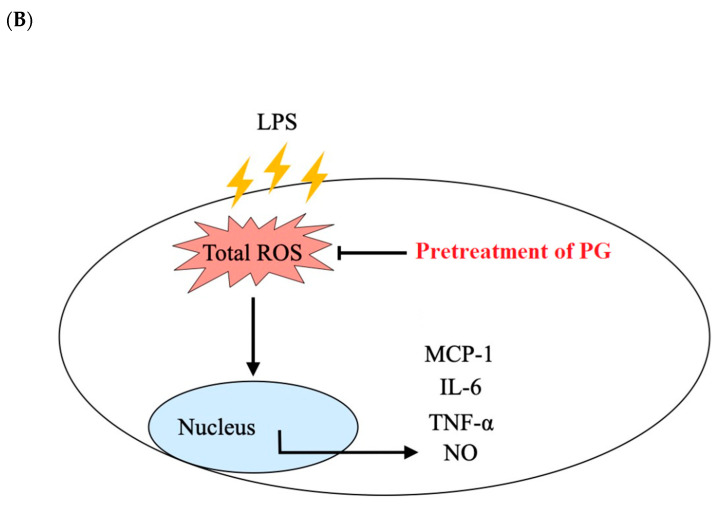
Graphical abstract of our experiments (**A**) and the potential therapeutic mechanism (**B**). In our macrophage-targeted prophylactic model, the pretreatment of purple shoot green tea (PG) effectively scavenge lipopolysaccharide (LPS)-induced reactive oxygen species (ROS) and nitric oxide (NO) through modulating cytokine expressions (MCP-1, IL-6 and TNF-α) that could serve as a natural product for immunomodulation. MCP-1: Monocyte chemoattractant protein-1; IL-6: Interleukin-6; TNF-α, tumor necrosis factor-α.

There were no significant inhibitory effects of the tea samples on the secretion of IL-6 or TNF-α while co-cultured with LPS in the inflammatory macrophage model. The extracts of TG, TB and PB also induced a slight increase in IL-6 expression, however, PG was the exception. MCP-1, also known as chemoattractant CCL2, recruits responding cells which leads to enhanced expression of pro-inflammatory cytokines. The pivotal role has been recognized in the pathogenesis of diverse infectious diseases, particularly during the post-COVID-19 era [6,7,8]. Given four teas could inhibit the downstream signaling transduction of MCP-1/CCL2 in the inflammatory model (Figure 9), tea extracts may develop as prophylactic strategies for the spread of infectious diseases. Apparently, the MCP-1 suppressing activity of PG results from the ROS scavenging effects of combined activities of flavonoids and proanthocyanidins, rather than being attributable only to simple phenolic compounds. In particular, our findings are consistent with the study of Mehany et al. [17]. It is interesting that the PG has the most powerful inhibition on the expression of MCP-1/CCL2 (Figure 8) without increasing secretions of TNF-α (Figure 6) and IL-6 (Figure 7) in the inflammatory model. The molecular mechanisms of tea extract effects on modulating the inflammatory cascades are more intricate than expected, and additional pro-inflammatory mediators may be involved and may not be limited to TNF-α, IL-6 and MCP-1/CCL2. Figure 11 illustrates the schematic diagram of the therapeutic mechanism of purple shoot green tea extract in LPS-stimulated macrophages. ROS-induced cytokines and chemoattractants involved in the pathogenesis of the inflammatory model are more complex than illustrated. LPS stimulates macrophages to secrete NO, TNF-α, IL-6 and MCP-1, recruiting more responding cells with abundant production of diverse inflammatory mediators. Pretreatment of PG effectively scavenges ROS to attenuate bacterial infection-related cytokine storms and disease severity that could serve as a prophylactic agent in the post-COVID 19 era.

Overwhelmed pro-oxidant effects of free radicals or ROS have been found to be balanced by the antioxidant effects along with anti-inflammatory properties of dietary tea polyphenols in habitual tea drinkers [19,20,27]. In addition, the direct scavenging effects of ROS could modulate the intracellular inflammatory signaling transduction of severe lung injuries [2]. Haghighatdoost et al. published a systematic review and meta-analysis of randomized clinical trials, indicating that green tea might reduce inflammatory mediators in patients with profound inflammation, such as TNF-α, C-reactive protein and IL-6, from tumors [22]. Although COVID-19 patients were not destined to suffer from cytokine storms during two weeks of hospitalization [9], tea polyphenols such as EGCG were found to be potent inhibitors of cytokine storms in the post-COVID-19 era [22,23,24]. Based on the excellent results in the prophylactic model, the anti-inflammatory effects of our tea extracts were not only attributed to NO-scavenging ability, but also contributed to the suppression of pro-inflammatory mediator (TNF-α, IL6 and MCP-1/CCL2). Not limited to the prophylactic model, our study illustrates that tea extracts inhibit inflammatory cascades by inhibiting the expression of MCP-1/CCL2 which may participate in downstream NO pathways in response to LPS stimulation [28]. The PG contained EGCG and flavonoids which can disrupt the transduction of NO, NFκB and MCP-1/CCL2 pathways; therefore, is considered as a dominant phytochemicals against LPS-stimulated inflammation. Flavonoids are categorized as plant-derived mast cell stabilizers and have the potential to downregulate cytokine storms in cases of COVID-19 [29]. In view of the pivotal roles of diverse phytochemicals in human health and disease, beneficial foods could be used as dietary or complementary therapy to prevent infection and strengthen immunity [30,31]. In light of the combined results of our inflammatory and prophylactic models, habitual consumption of PG is associated with lower risks of oxidative stress-induced inflammatory cytokine cascades that serve as a healthy natural product.

## 5. Conclusions

Our experimental model of LPS-stimulated macrophages demonstrated that PG exerts robust effects on modulating LPS-induced cytokine expression of MCP-1, IL-6 and TNF-α through scavenging free radicals and NO. In light of the prophylactic effects on LPS-related inflammation, habitual consumption of PG effectively scavenges ROS to modulate cytokine cascades that could serve as a functional food for immunomodulation.

## Figures and Tables

**Table 1:** The comparisons of total polyphenols, flavonoids, condensed tannins and proanthocyanidins between various tea extracts.

Tea Samples	Total Phenolics (mg gallic acid eq./g)	Total Flavonoids (mg catechin eq./g)	Condensed Tannins (mg catechin eq./g)	Proanthocyanidins (mg cyanidin chloride eq./g)
TB	238.70 ± 0.26	54.09 ± 1.77	113.70 ± 5.83	7.99 ± 0.06
TG	321.95 ± 10.58	64.82 ± 0.83	233.67 ± 6.61	10.88 ± 0.46
PB	291.90 ± 4.69	68.00 ± 1.16	123.39 ± 4.92	15.98 ± 0.35
PG	371.28 ± 3.83	86.37 ± 1.46	234.67 ± 10.10	24.81 ± 0.75

T: Taiwan Tea Experiment Station No. 12; P: purple shoot tea; G: green tea; B: black tea. Data are expressed as means ± S.E. of different independent experiments (*n* = 3). All *p* values < 0.05. Note that PG contains the highest levels of phenolics, flavonoids, tannins and proanthocyanidins.

**Table 2:** Quantitative analysis of diverse types of constituents in various tea extracts.

Concentration in Various Tea Extracts (µg/mg)				
Constituents	TG	TB	PG	PB
GA	4.28 ± 0.02	27.14 ± 0.97	6.07 ± 0.14	30.45 ± 0.55
GC	61.64 ± 0.30	N.D.	57.54 ± 0.94	N.D.
EGC	97.33 ± 0.51	51.36 ± 2.47	132.97 ± 1.6	61.70 ± 2.19
Catechin	12.98 ± 0.03	7.53 ± 0.67	14.90 ± 0.13	9.21 ± 0.32
Caffeine	32.14 ± 0.59	39.68 ± 3.63	48.83 ± 3.31	54.16 ± 1.27
EC	17.27 ± 0.09	8.43 ± 0.71	26.56 ± 0.56	13.12 ± 1.10
EGCG	119.97 ± 2.40	24.90 ± 1.19	134.86 ± 8.05	29.23 ± 0.93
GCG	82.52 ± 1.64	8.26 ± 0.26	70.62 ± 1.35	11.03 ± 1.10
ECG	17.92 ± 0.38	13.48 ± 0.08	22.12 ± 0.22	15.16 ± 0.26
CG	2.90 ± 0.18	N.D.	3.42 ± 0.30	N.D.

Note that PG contains the highest levels of EGC, catechin, EC, EGCG, ECG and CG. N.D. = non-detected; GA = gallic acid; GC = gallocatechin; EGC = epigallocatechin; EC = epicatechin; EGCG = epigallocatechin gallate; GCG = gallocatechin gallate; ECG = epicatechin gallate; CG = catechin gallate; T: Taiwan Tea Experiment Station No. 12; P: purple shoot tea; G: green tea and B: black tea. All *p* values < 0.05 using Tukey—Kramer’s test.

## Data Availability

All data used to support the findings of this study are available from the corresponding author, Jia-Feng Chang, upon reasonable request. Corresponding author’s email: cjf6699@gmail.com.
